# Exosomal lncRNAs as regulators of breast cancer chemoresistance and metastasis and their potential use as biomarkers

**DOI:** 10.3389/fonc.2024.1419808

**Published:** 2024-08-01

**Authors:** Sugela Susana Blancas-Zugarazo, Elizabeth Langley, Alfredo Hidalgo-Miranda

**Affiliations:** ^1^ Cátedras CONAHCYT (Consejo Nacional de Humanidades Ciencia y Tecnología) - Laboratorio de Genómica del Cáncer, Instituto Nacional de Medicina Genómica (INMEGEN), Mexico City, Mexico; ^2^ Laboratorio de Cáncer Hormono Regulado, Instituto Nacional de Cancerología (INCAN), Mexico City, Mexico; ^3^ Laboratorio de Genómica del Cáncer, Instituto Nacional de Medicina Genómica (INMEGEN), Mexico City, Mexico

**Keywords:** breast cancer, lncRNA, exosomes, metastasis, chemoresistance, cancer biomarkers

## Abstract

Breast cancer is the most common cancer in women and the leading cause of female deaths by cancer in the world worldwide. Hence, understanding the molecular mechanisms associated with breast cancer development and progression, including drug resistance and breast cancer metastasis, is essential for achieving the best management of breast cancer patients. Cancer-related long noncoding RNAs have been shown to be involved in the regulation of each stage of breast cancer progression. Additionally, exosomes are extracellular microvesicles that are central to intercellular communication and play an important role in tumorigenesis. Exosomes can be released from primary tumor cells into the bloodstream and transmit cellular signals to distant body sites. In this work, we review the findings regarding the cellular mechanisms regulated by exosomal lncRNAs that are essentials to chemoresistance development and metastasis of breast cancer. Likewise, we evaluate the outcomes of the potential clinical use of exosomal lncRNAs as breast cancer biomarkers to achieve personalized management of the patients. This finding highlights the importance of transcriptomic analysis of exosomal lncRNAs to understand the breast cancer tumorigenesis as well as to improve the clinical tests available for this disease.

## 1 Introduction

### 1.1 Breast cancer

Breast cancer (BC) represents a worldwide public health challenge due to its high associated mortality and morbidity rates. The 5-year survival rate of patients with metastatic BC after treatment with adjuvant therapy is less than 30% (1). According to GLOBOCAN 2020 statistics, the number of new cases of BC worldwide was 2.3 million (11.5%), and the number of deaths was 666 103 (6.8%) (2). In women, the BC is the most diagnosed cancer in 159 of 185 countries and is the leading cause of cancer death in 110 of 185 countries (3). BC incidence is highly correlated with human development (4). Thus, incidence rates are 88% higher in developed than in developing countries (55.9 and 29.7 per 100,000, respectively). However, women living in developing countries have 17% higher mortality rates compared with women in developed countries (15.0 and 12.8 per 100,000, respectively) (3). The elevated incidence rates in higher Human Development Index (HDI) countries are associated with BC risk factors (4). The HDI promote a longstanding higher prevalence of reproductive and hormonal risk factors and lifestyle risk factors, as well as increased detection through organized or opportunistic mammographic screening (3).

According with the data, the average age of women deaths associated to BC is lower in developing countries than in countries with high in-come. That is associated to several factor including the late diagnostic, the poor health services and lack of treatments. Thus, in developing countries, more than half of BC is in women under 50, shortening life expectancy in those countries by a decade (4). Almost two-thirds of the deaths in 2020 were recorded in less-developed regions. In developed countries, over 80% of BC patients present an overall survival of 5-year, in contrast to developing regions with 5 years survival less than 50% (3, 4). The observed BC survival profile is associated with later diagnostics that are common in less developed regions of the world, with over half of breast cancers being locally advanced or metastatic at diagnostic (4). Thereby, establishing primary prevention programs for BC remains as a challenge and all approaches to screening tests and early diagnoses are essential, mainly in countries with developing economies (3).

BC usually refers to a group of diseases with biological subtypes that reflect different molecular profiles and clinicopathological characteristics. In addition to histological subtypes, immunohistochemical classification has divided BC into 5 main molecular subtypes: luminal A (estrogen receptor [ER] +, progesterone receptor [PR] +, human epidermal growth factor receptor 2 [HER2] –, Ki-67 low); luminal B HER2 – (ER+, PR+, HER2-, Ki-67 high); luminal B HER2+ (ER+, PR+, HER2+, Ki-67 high); HER2 (ER-, PR-, HER2+); and basal (triple negative [TNBC], ER-, PR-, HER2-), which are related to clinical outcome (5). Additionally, gene expression profiling and molecular diagnosis have significantly impacted the management of BC. Groups of genes have been identified as biomarkers that help predict disease prognosis and estimate the risk of metastasis, tumor recurrence, response to therapy, and clinical decision making. Furthermore, they have helped guide clinical test development for patient follow-up (5, 6).

### 1.2 Exosome biology and cancer

Exosomes are a class of lipid bilayer-enclosed extracellular vesicles (EVs) that are devoid of intracellular organelles but contain all known molecular constituents within a cell (7, 8). The exosome size ranges from 30 nm to 150 nm (9–11), and exosomes are constantly released by most eukaryotic cells, including platelets, mast cells, dendritic cells, astrocytes, B and T cells, and cancer cells. *In vivo*, exosomes are broadly observed in numerous body fluids, such as blood, serum, saliva, amniotic fluid, and breast milk (12). In particular, exosomes enclose a wide range of molecules, including proteins, lipids, and other metabolites (7, 13). Furthermore, they also contain single-stranded (14) and double-stranded DNA (15) and different kinds of RNA, such as mRNAs, tRNAs, rRNAs, miRNAs, siRNAs, circRNAs (16), lncRNAs (17), snoRNAs, snRNAs (18) and piRNAs (19). In addition, genomic, mitochondrial, and plasmid DNA have all been identified within exosomes (20–22).

Exosomes are important mediators of intercellular communication and are involved in several physiological and pathological processes (10). Intriguingly, tumor cells secrete approximately 10-fold more exosomes than do normal cells (23), and tumor-derived exosomes (TDEs) play important roles in different stages of cancer progression (24, 25). TDEs can enhance angiogenesis, invasion, and migration, promote premetastatic niche establishment and confer chemoresistance (26–29). The high heterogeneity of TDEs likely reflects the phenotypic state of tumor cells that generate exosomes (30, 31). Thus, TDE analysis provides a robust method for monitoring cancer progression and further guiding clinical decisions and treatment strategies (32). The stability of exosomes in most body fluids and the diversity of their cargo, which reflects the status of the parental cells, make them promising candidates for developing new approaches for cancer diagnosis (33, 34). An advantage of TDE analysis is that their lipid bilayers stabilize and protect macromolecules against enzymatic activity existing in biofluids, unlike other biomarker assays requiring fresh biofluid (35, 36). This allows them to be stored for an extended period (35, 36), thereby greatly increasing their clinical applications while reducing the cost of short-term sample storage (12, 37). TDEs provide a promising platform for cancer prognosis, diagnosis, and treatment follow-up in precision and personalized medicine (12). EVs can be purified, and their isolated materials can be further analyzed using next-generation sequencing (NGS), real-time PCR, digital PCR, and bioinformatics for screening and early detection of cancer (38). The previous describe the advantages that exosomes offer in the development of new clinical tests for the diagnosis and monitoring of breast cancer, however it cannot be ignored that there is still a long way to go in the sense of reaching standardized methods for the purification, characterization, and analysis of exosomes with adequate quality for use in the clinic.

### 1.3 LncRNAs biology and cancer: perspectives in BC diagnostic

Noncoding RNAs (ncRNAs) are classified by their size, the longest of which are long noncoding RNAs (lncRNAs) with more than 200 nucleotides. LncRNAs are transcribed by RNA polymerase II, and similar to mRNAs, they have a 5’-cap and 3’ poly-A tail (39, 40). According to their chromosomal positions, lncRNAs are classified as antisense, intronic, divergent, intergenic, promoter associated, transcription start site associated, or enhancer (39). LncRNAs have different functions according with their cellular localization. In the nucleus, they are involved in epigenetic and transcriptional regulation, whereas in the cytoplasm, they are associated with posttranscriptional regulation, including mRNA stability and protein translation, and can act as competitive endogenous RNAs (ceRNAs) (39).

It is widely known that lncRNAs play pivotal roles in each state and process of cancer development (39, 40). Since the discovery of metastasis-associated lung adenocarcinoma transcript 1 (MALAT1) in 2003 (41), many other cancer-associated lncRNAs have been identified, and their expression can be deregulated. Among the most characterized lncRNAs associated with cancer development and drug resistance are H19 (42), X-inactive-specific transcript (XIST) (43), and homeobox (HOX) transcript antisense RNA (HOTAIR) (44). The role of lncRNAs in cancer development has been established for a number of these molecules, and four main mechanisms of action have been proposed, including acting as signals for transcriptional regulation, acting as decoys that recruit binding partners away from their other targets, acting as scaffolds bringing together multiple biomolecules, and acting as guides directing the targeting of molecular complexes (39, 40).

In this context, due to the lncRNAs are regulators of diverse oncogenic processes, they could be cancer biomarkers. Particularly in BC, the current screening and diagnostic methods for BC are mammography, ultrasound, MRI, and biopsy. However, the prognostic potential of these methods to predict the BC course, including metastasis, is still very limited (45, 46). Likewise, it is important to identify screening biomarkers to measure the BC risk before the tumor onset (45). BC conventional serum biomarkers are carcinoembryonic antigen (CEA) and cancer antigen 15–3 (CA15–3), however their clinical use is limited by their low sensitivity and specificity (45). In this context, aberrant expression of lncRNAs has been observed in many diseases including cancer and lncRNAs possess a high degree of specificity for tissue type and disease, becoming ideal candidates for cancer diagnosis (45). There are some approaches that analyze the use of levels of lncRNAs in circulation as biomarkers of BC. Furthermore, they analyze the potential of measuring the levels of exosomal lncRNAs. In many of these studies, the certainty of using exosome lncRNAs has been compared versus the use of CEA and CA15–3, with promising results that indicate greater sensitivity and specificity when the 3 biomarkers are used (47). However, the current studies investigating the use of EV ncRNA as biomarkers in BC have been focused on discovery and initial technical validation (46) and the clinical implementation for the use of exosomal lncRNAs needs more research to develop standardized methods that reduce the variability between results. It is necessary to resolve issues such as pre-analytical variables (sample type, storage methods, environmental factors, etc); the methods to be used for exosomal RNA isolation and measurements; and standardization of methodologies to normalize the procedures and reduce inter-laboratory and inter-user variability (45).

## 2 Advantages of tumor-derived exosomes for use in genomic and transcriptomic analyses for biomarker identification

RNA-seq technology has revealed that all forms of RNA can be detected in exosomes and can be useful for cancer analysis (48, 49). Using high-throughput sequencing technologies, researchers have found that exosomes contain different RNA populations, including circRNAs (34), lncRNAs (35, 50), mRNAs (51, 52), miRNAs (53, 54), mRNA fragments (55), piRNAs, and fragments of numerous noncoding RNAs, including tRNAs and rRNAs (35, 56, 57). Specifically, several exosomal miRNAs were recently described as being diagnostic for lung cancer (58, 59). Additionally, increasing evidence has shown that circRNAs are highly enriched and stable in exosomes (49). Compared with circulating tumor DNA (ctDNA) assays, circulating nucleic acids from exosomal sources could increase the number of mutant copies accessible for sampling (34, 60). This suggests that exosomal RNA may increase the potential for detecting mutations in blood samples, particularly when very few copies of ctDNA are available during the early stages of disease (34, 60). For clinically reliable and suitable NGS analyses in the future, standardization and clinical verification are necessary. Currently, exploring biomarkers in TDEs has shown great potential for the diagnosis, monitoring, and treatment of cancer patients but still has obvious limitations (32). However, the development of standardized methods for the isolation and identification of TDEs, as well as the use of NGS techniques, could be an excellent tool for diagnosing, monitoring, and guiding cancer therapy.

There are several examples in which RNA-seq technology has been used to identify and describe the role of exosomal ncRNAs associated with BC. Jenjaroenpun and collaborators (2013) were the first to analyze the transcriptomes of exosomes derived from the human BC cell lines MDA-MB-231 and MDA-MB-436 (61). Exosomes contain many classes of RNA, the most common of which is fragmented rRNA. Importantly, the analysis of exosomal RNA reflected the RNA of the donor cells, and several noncoding transcripts were unique to the MDA-MB-231 and MDA-MB-436 cells. In this case, RNA-seq analysis was able to distinguish exosomal RNA delivered by highly metastatic cells (MDA-MB-231) from that delivered by less metastatic cells (MDA-MB-436) (61). In a subsequent exploratory study, RNA sequencing analysis was carried out on serum exosomes derived from one healthy female and two BC patients, followed by comparative analysis with reference data, Gene Ontology (GO), and Kyoto Encyclopedia of Genes and Genomes (KEGG) pathway enrichment analyses. Based on these methodologies, they identified five upregulated and six downregulated exosomal lncRNAs as potential biomarkers (62). They proposed the lncRNAs VIM-AS1 (upregulated; with 35 predicted target miRNAs), SNHG8 (downregulated; with 12 predicted target miRNAs), and ELDR (downregulated; with 24 predicted target miRNAs) (62) as possible diagnostic biomarkers. In a more recent study, with the analysis of the Cancer Genome Atlas (TCGA) database and the use of RNA-seq technology, the expression profiles of lncRNAs in EVs from BC patient plasma were analyzed. In this work, the authors identified five lncRNAs in tissue and plasma EVs that could be developed as biomarkers for BC. Four of these lncRNAs (C15orf54, AL157935.1, LINC01117, and SNHG3) were proposed as diagnostic markers for BC lesions, although the plasma EVs from patients were not significantly different. The last lncRNA, AL355974.2, was proposed to be an independent protective prognostic factor after survival analysis (63). These three studies previously described are excellent approaches in the use of NGS techniques and bioinformatic analysis for the massive search of new exosomal biomarkers for BC. However, the results of these studies require further validation with a significant number of exosome samples from BC patients. The study of Jenjaroenpun and cols. was carried out with BC cell lines (61). In the second study, only the exosomal samples of serum from two patients were sequenced (62) and in the third, the results of the levels in EVs of the molecules under study, did not show significant differences between BC patients and healthy ones (63), so they proposed the lncRNAs founded as diagnostic biomarkers of BC only for tissue.

Similarly, there have been several initiatives to develop exosome-based databases for biomarker discovery. These include ExoBCD (64) and exoRBase (65). Using exoRBase, the exosomal sequencing data of BC patients and normal controls were analyzed to identify a ceRNA regulatory network with differential expression profiles from exosomes. Overall, 42 mRNAs, 43 circRNAs, and 26 lncRNAs were found to be differentially expressed (65). In another study using the ExoBCD database, the authors analyzed exosomal lncRNAs and microenvironment interactions in BC. They identified 15 exosome-related differentially expressed lncRNAs that correlated with BC prognosis, and further bioinformatic analysis allowed them to construct a risk model to predict survival outcome. This exosome-related lncRNA risk model could provide a tool to estimate prognosis and immune cell infiltration in BC patients, providing important information for immunotherapeutic decisions (66). The last is an excellent example of the potential of using data stored in platforms such as ExoBCD and exoRBase, so it is necessary to increase the data from these platforms and thus increase their potential in the bioinformatic analysis of the content of exosomes, their function and their possible use as diagnostic and monitoring markers for BC.

## 3 Exosomal lncRNAs mediate chemoresistance in breast cancer

One of the main obstacles in cancer management is the development of drug resistance during chemotherapy treatment. Basically, there are two types of drug resistance: intrinsic or natural resistance and acquired resistance, in which therapeutic effectiveness is attenuated over time (67). However, Vasan and colleagues (2019) explained that many tumors are resistant or become resistant due to combinations of each type of resistance (68). Specifically, in BC, the use of different chemotherapies for each of the molecular types of tumors results in drug resistance in a high percentage of BCs (69). Approximately 30%-40% of BC patients are resistant to endocrine therapies and develop metastatic conditions. In addition, modifications of HER receptor signaling have been reported to play a substantial role in the development of BC drug resistance (69). In this context, the role of exosomal ncRNAs in the development of drug resistance has already been reviewed (69); here, we focus only on exosomal lncRNA functions in chemoresistance, including the most current data and summarized in the **Table 1**.

**Table 1:** Exosomal lncRNAs associated to chemoresistance in BCC and BC patients.

Drug resistance	LncRNA	Exosome source	Molecular subtype of BC or BCC	Level of exosomal lncRNA	Reference
Tamoxifen	UCA1	MCF-7 cells	ER+	High	Xu et al. (2016) (70)
	HOTAIR	Serum	ER+	High	Tang et al. (2019) (71)
Trastuzumab	AGAP2-AS1	SKBR-3 and BT474 cells	HER2+	High	Zheng et al. (2019) (72)
	OIP5-AS1	SKBR-3 and BT474 cells	HER2+	High	Yu et al. (2021) (73)
	OIP5-AS1	Serum	HER2+	High	Yu et al. (2021) (73)
Doxorubicin	H19	MCF-7 and MDA-MB231 cells	ER+ and TNBC cells	High	Wang et al. (2020) (74)
		Serum	No specify	High	Wang et al. (2020) (74)
	MALAT1	MCF-7 cells	ER+	High	Tao et al. (2022) (75)
	SNHG14	MCF-7 cells	ER+	High	Wan et al. (2023) (76)
Docetaxel	LINC00667	MDA-MB-231 cells	TNBC	High	Li et al. (2022) (77)
Paclitaxel	NEAT1	SKBR3 cells	HER2+	High	Wei et al. (2023) (78)

BCC, Breast cancer cells; BC, Breast cancer.

### 3.1 Tamoxifen resistance

**Figure 1:**
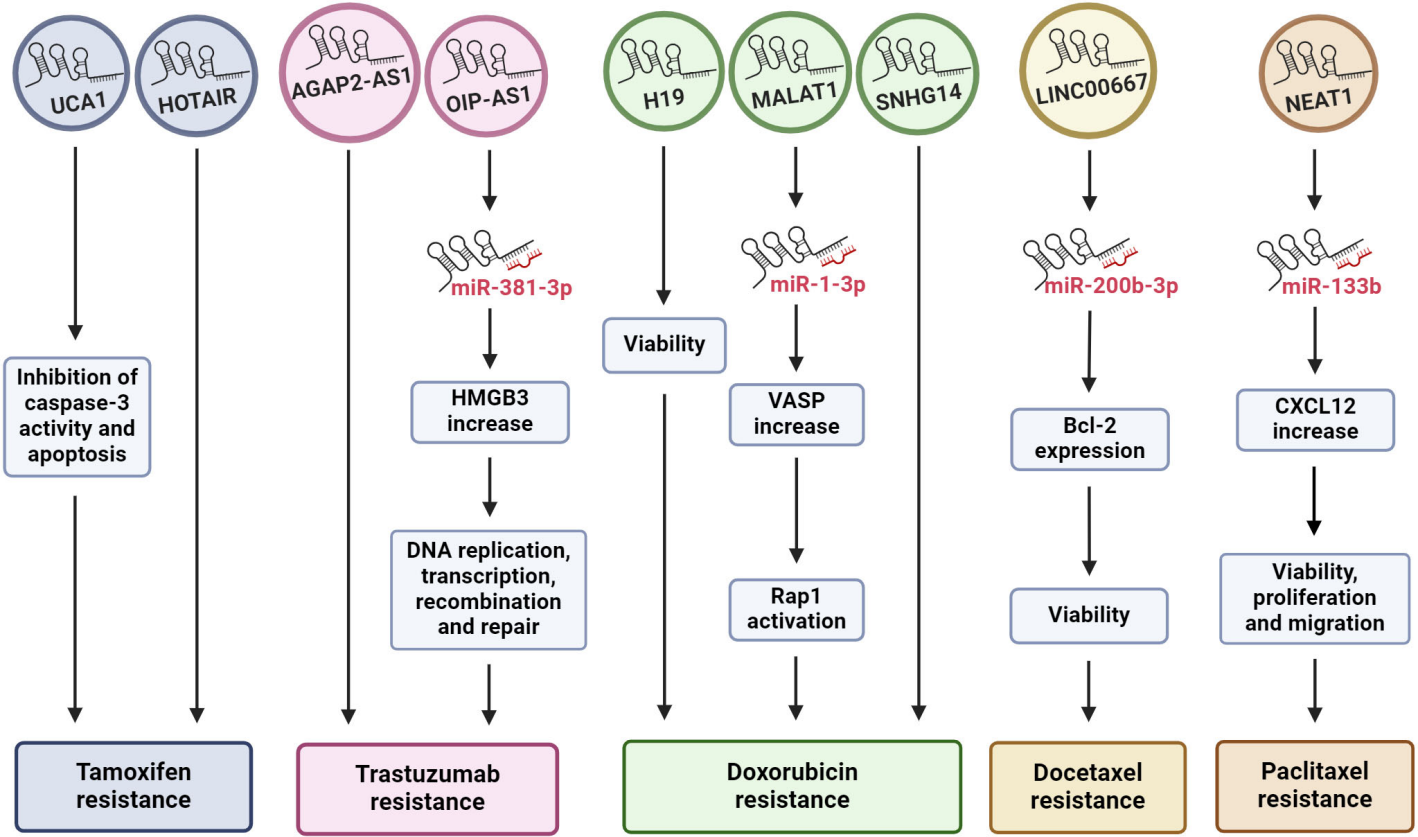
Exosomal lncRNAs regulate drug resistance. The diagram shows how the effect of some lncRNAs released by BC cells, mediated by exosomes, can promote drug resistance through different cell pathways, including in many cases the sponging of miRNAs. The chemoresistance to each specific drug can be modulated by more than one exosomal lncRNA. HMGB3, high mobility group box 3; VASP, vasodilator-stimulated phosphoprotein; Rap 1, Ras-related protein 1; CXCL12, C-X-C motif chemokine ligand 12.

Tamoxifen is a nonsteroidal synthetic selective estrogen receptor modulator (SERM) that inhibits estrogen receptor (ER) activity in the breast and is a widely used therapeutic agent for BC patients with ER-positive tumors (79). However, endocrine therapy resistance occurs in a significant number of patients. The oncogenic function of the lncRNA urothelial carcinoma-associated 1 (UCA1) in BC has been described previously (80, 81). In an *in vitro* study in the ER-positive human BC cell line MCF-7 and a tamoxifen-resistant derivative of these cells (LCC2), Xu and collaborators (2016) explored the role of UCA1 in tamoxifen resistance (70). They showed that UCA1 is highly expressed not only in LCC2 cells but also in exosomes released from LCC2 cells compared with exosomes from tamoxifen-sensitive MCF-7 cells. Moreover, incubation with exosomes derived from LCC2 cells increased the viability of MCF-7 cells treated with tamoxifen, reducing apoptosis by decreasing cleaved caspase-3 activity (**Figure 1**). However, the capacity of LCC2 exosomes to induce tamoxifen resistance was inhibited when their cargo contained impaired UCA1. Thus, UCA1 transfer mediated by exosomes can significantly increase tamoxifen resistance in ER-positive MCF-7 cells (70).

As mentioned above, HOTAIR is a widely studied lncRNA due to its pivotal role in many types of malignant tumors (82–84), including BC, where HOTAIR is highly expressed (85, 86). Additionally, studies have shown that there are greater levels of HOTAIR in exosomes from patients with bladder (87) and cervical (88) cancer than in those from healthy individuals. Moreover, exosomal HOTAIR has been suggested to be a good prognostic and diagnostic biomarker in laryngeal squamous cell carcinoma (89). Tang and coworkers (2019) (71) carried out a study on BC patients who were followed for approximately 6 years to evaluate the diagnostic and prognostic value of serum exosomal HOTAIR. Serum samples from 15 healthy individuals, 15 BC patients treated surgically, 25 patients who received neoadjuvant chemotherapy (anthracycline + taxane + cyclophosphamide regimen) before surgery, and another 25 patients who received tamoxifen treatment after surgery were analyzed. Additionally, 20 BC tissue samples were collected. They found that BC patients had high serum exosomal HOTAIR levels, which decreased 3 months after surgery; thus, exosomal HOTAIR is produced by primary tumor cells and released by exosomes into the bloodstream (71). Furthermore, exosomal HOTAIR levels increased with culture time in the human BC cell lines MDA-MB-231 (triple negative) and MCF-7. In a xenograft assay in which nude mice were injected with BC cells, the expression of serum exosomal HOTAIR in the mice was notably greater than that in the mock control group. The authors also explored the diagnostic and prognostic value of serum exosomal HOTAIR and found that high expression of exosomal HOTAIR led to worse disease-free survival and overall survival, indicating that exosomal HOTAIR could be a good diagnostic and prognostic biomarker. When they analyzed the response to chemotherapy, they observed a correlation between poor response and the overexpression of exosomal HOTAIR. In the HOTAIR high-expression group (n=14), 6 patients achieved a partial response (PR), and 8 achieved stable disease (SD). Conversely, in the low-expression group (n=11), 9 patients achieved a PR, and 2 achieved SD. Similarly, there was a better response to tamoxifen in the low exosomal HOTAIR expression group, as only 1 patient (of 11) experienced BC recurrence, while in the high-expression group, 6 (of 13) experienced recurrence (71). This finding implies that high HOTAIR expression is associated with a poor response to endocrine therapy with tamoxifen and that this effect could be modulated by increased levels of exosomal HOTAIR in serum derived from primary BC tumors and released into the bloodstream (**Figure 1**). Therefore, the authors suggest that serum exosomal HOTAIR is a prognostic and diagnostic biomarker for BC patients and may be useful for making therapeutic decisions in terms of endocrine therapy (71). Furthermore, the expression of exosomal HOTAIR in plasma was positively correlated with the HER2 status of BC patients, supporting the possible use of HOTAIR as a BC prognostic biomarker (90).

### 3.2 Trastuzumab resistance

Trastuzumab is an antibody against HER2 that is commonly used as a treatment for HER-2-positive BC. Trastuzumab treatment improves the clinical prognosis of patients, prolonging overall survival in adjuvant and metastatic settings (91). Recently, the role of AGAP2 antisense RNA 1 (AGAP2-AS1) in trastuzumab resistance *in vitro* was explored. The lncRNA AGAP2-AS1 has an oncogenic function in human non-small cell lung cancer (92, 93) and gastric cancer (94). Zheng and colleagues (2019) (72) induced trastuzumab resistance in the HER2-positive BC cell lines SKBR-3 and BT474. AGAP2-AS1 is upregulated in trastuzumab-resistant SKBR-3 and BT474 cells (SKBR3-TR and BT474-TR, respectively), but silencing AGAP2-AS1 reversed trastuzumab resistance. The authors demonstrated that trastuzumab-resistant cell lines release exosomes loaded with AGAP2-AS1 in a manner dependent on RNA-binding hnRNPA2B1, which mediates the packaging of RNAs into exosomes. Moreover, these exosomes can induce trastuzumab resistance in sensitive cells, an effect that is dependent on exosomal AGAP2-AS1 (**Figure 1**). The authors suggested that knockdown of AGAP2-AS1 may be helpful for improving the clinical outcome of HER2-positive BC patients and could serve as a therapeutic target (72).

Moreover, the lncRNA OPA-interacting protein 5 antisense transcript 1 (OIP5-AS1) plays many oncogenic roles in multiple cancers (95), including BC (96). In an *in vitro* assay using the same trastuzumab-resistant cells, SKBR3-TR and BT474-TR, OIP5-AS1 was elevated, and resistance was dependent on OIP5-AS1 (73). Like AGAP2-AS1, OIP5-AS1 is released from resistant cells via exosomes, and these exosomes can be absorbed by trastuzumab-sensitive cells and induce cellular resistance. Mechanistically, OIP-AS1 acts as a sponge for miR-381–3p. miR-381–3p targets high mobility group box 3 (HMGB3), which can regulate DNA replication, transcription, recombination, and repair (**Figure 1**) (97). Therefore, HMGB3 silencing can inhibit cell growth and progression in BC (98). The ability of OIP5-AS1 to induce trastuzumab resistance was established in a murine xenograft model in which the transfer of exosomal OIP5-AS1 induced trastuzumab resistance *in vivo*. Moreover, exosomal OIP5-AS1 was dysregulated in the serum of BC patients and might be a promising diagnostic biomarker for trastuzumab resistance (73).

### 3.3 Doxorubicin resistance

LncRNA H19 functions as an oncogene in numerous cancer types, including gastric (99), colorectal (100), pancreatic (101) and BC, to the extent that plasma H19 has been proposed as a diagnostic and prognostic biomarker for BC (47, 102). The role of H19 in the development of doxorubicin resistance in BC cells has recently been explored. Doxorubicin (DOX) is an anthracycline used as a broad-spectrum anti-neoplastic drug and is included in first-line adjuvant BC treatment (103). As mentioned above, the human BC cell lines MCF-7 and MDA-MB-231 are molecularly different. MCF-7 is a hormone-responsive human invasive breast adenocarcinoma that represents a luminal A subtype, while MDA-MB-231 is a basal subtype that does not express hormone receptors and contains the mutant p53 protein. Wang and collaborators (2020) (74) induced doxorubicin resistance in both cell types (MCF-7/DOX and MDA-MB231/DOX). They observed an increase in H19 expression in DOX-resistant BC cells compared with the corresponding parental cells. Additionally, they established that DOX resistance was an H19-dependent event since H19 suppression significantly lowered DOX resistance by decreasing cell viability and inducing apoptosis. Furthermore, DOX resistance is induced in sensitive cells by exosomal H19 (**Figure 1**). In BC patients, serum exosomal H19 levels are elevated in DOX-resistant patients (46 of 82 total patients). The sensitivity and specificity of exosomal H19 were 75% and 65.2%, respectively, for the prediction of BC resistance to therapy (74). Moreover, doxorubicin resistance occurred in both the basal and hormone receptor-positive subtypes.

Small nucleolar RNA host gene 14 (SNHG14) plays a pivotal role in the carcinogenesis of several malignant tumors, such as BC, by regulating cell proliferation, migration, invasion, and chemoresistance (104). Using RNAseq technology, the differential expression profiles of lncRNAs in DOX-resistant MCF7 cells were determined. These results and the analysis of gene expression omnibus (GEO) datasets allowed researchers to identify the altered expression of SNHG14, which was increased in BC tissues and in MCF7/DOX cells (76). In addition, SNHG14 levels were greater in purified exosomes from MCF7/DOX cells than in those from parental cells. Additionally, exosomal SNHG14 could be transmitted from drug-resistant cells to drug-sensitive cells after coincubation of DOX-sensitive cells with purified MCF7/DOX exosomes (**Figure 1**). However, the role of SNHG14 in cellular DOX resistance must be further explored in detail (76).

Interestingly, although the MCF-7 and MDA-MB-231 cell lines have distinct molecular profiles, they show similar tamoxifen and doxorubicin resistance responses. In both cell lines, tamoxifen resistance is modulated by HOTAIR, and doxorubicin resistance is modulated by H19. These data imply a molecular mechanism of chemoresistance that is dependent on the treatment and not the molecular profile of BC cells, at least in terms of the effects associated with HOTAIR and H19.

### 3.4 Docetaxel and paclitaxel resistance

Docetaxel and paclitaxel are two taxanes widely used in clinical cancer treatment. Both drugs bind to the β-subunit of tubulin, stabilizing microtubules and interfering with natural cytoskeleton dynamics, thereby inhibiting mitosis. Paclitaxel acts at the M/A-phase transition, whereas docetaxel is primarily active in G2/M-phase. Therefore, these drugs inhibit proliferation and cell viability, induce apoptosis, and have antiangiogenic effects (105). Docetaxel has been used for treating various cancers, including triple-negative breast cancer (TNBC). Although docetaxel arrests the cell cycle in cancer cells, acquired resistance is an important obstacle for the treatment of TNBC (105, 106). Docetaxel resistance appears to be regulated by lncRNAs, such as LINC00667. Studies have shown that LINC00667 expression is elevated in BC tissues (107) and that this lncRNA promotes the proliferation and migration of BC cells (108). LINC00667 expression is elevated in exosomes derived from MDA-MB-231 cells resistant to docetaxel compared to exosomes from docetaxel-sensitive cells. In addition, LINC00667 can be transmitted via exosomes from docetaxel-resistant to docetaxel-sensitive cells. Furthermore, exosomal LINC00667 induced docetaxel resistance in sensitive cells through the upregulation of antiapoptotic Bcl-2. In this regard, LINC00667 seems to function as a ceRNA to sponge miR-200b-3p, resulting in increased expression of Bcl-2 (**Figure 1**) (77).

Similarly, high expression of the lncRNA nuclear paraspeckle assembly transcript 1 (NEAT1) in SKBR3 BC cells promotes migration, proliferation, and paclitaxel resistance (78). NEAT1 downregulation decreased paclitaxel resistance, cell migration and cell proliferation. This effect is mediated by the release of miR-133b, which in turn downregulates C-X-C motif chemokine ligand 12 (CXCL12), a well-known inducer of cell survival, proliferation, migration, and drug resistance (**Figure 1**) (109). Moreover, paclitaxel-resistant SKBR3 cells produce exosomes loaded with high levels of NEAT1, and these exosomes are able to induce paclitaxel resistance, cell migration and growth in paclitaxel-sensitive BC cells. In a xenograft mouse model, knockdown of NEAT1 decreased cancer progression and improved the response to paclitaxel (78).

## 4 Exosomal lncRNAs promote metastasis

The most common sites of BC metastasis are the lung, bone, lymph nodes, liver, and pleura, with the highest incidence in the lung (110). Therefore, Feng and collaborators (2019) (111) used high-throughput sequencing to analyze lncRNA expression profiles in lung fibroblasts treated with exosomes derived from MDA-MB-231 BC cells compared with exosomes from normal epithelial MCF-10A cells. Exosomes from BC cells increase the proliferation and migration of lung fibroblasts (the WI-38 and HFL1 cell lines). They identified many lncRNA expression abnormalities that could be associated with alterations in the lung microenvironment and metastasis, including 141 upregulated lncRNAs and 98 downregulated lncRNAs in both lung fibroblast lines (111).

More than 30% of patients with metastatic BC develop brain metastasis (112). Additionally, BC is the second most common cause of brain metastases, following lung cancer, with the highest incidence in BC patients with HER2-positive and TNBC subtypes (113). In this context, the lncRNA expression profile associated with brain metastasis from BC patients showed that XIST was downregulated in brain tumors (114). Moreover, XIST is downregulated in the BC cell lines MDA-MB-231-BrM2a and SKBrM3 (derived from MDA-MB-231 and SKBR3, respectively), which preferentially metastasize to the brain. Thus, XIST plays a protective role in brain metastasis development. This finding was supported in a mouse xenograft model in which XIST silencing promoted brain metastasis. Specific knockout of XIST in mouse mammary glands accelerated primary tumor growth as well as metastasis to the brain. Additionally, XIST downregulation stimulated epithelial–mesenchymal transition (EMT) and activated the c-Met pathway by upregulating the MSN gene. Loss of XIST also augmented the secretion of exosomal miRNA-503, leading to reprogramming of microglia in the brain and triggering M1–M2 polarization of microglia. M1-M2 macrophage conversion upregulated the expression of immunosuppressive cytokines in microglia that suppressed T-cell proliferation. The authors searched an FDA-approved drug library and showed that fludarabine selectively inhibits the growth of XIST-low tumor cells. Additionally, they observed that fludarabine blocked brain metastasis in a mouse model. Furthermore, the loss of XIST promoted brain metastasis in BC by altering the tumor cell microenvironment and stimulating EMT. This suggested that fludarabine could be a good therapeutic agent for the prevention and treatment of brain metastasis (114).

**Figure 2:**
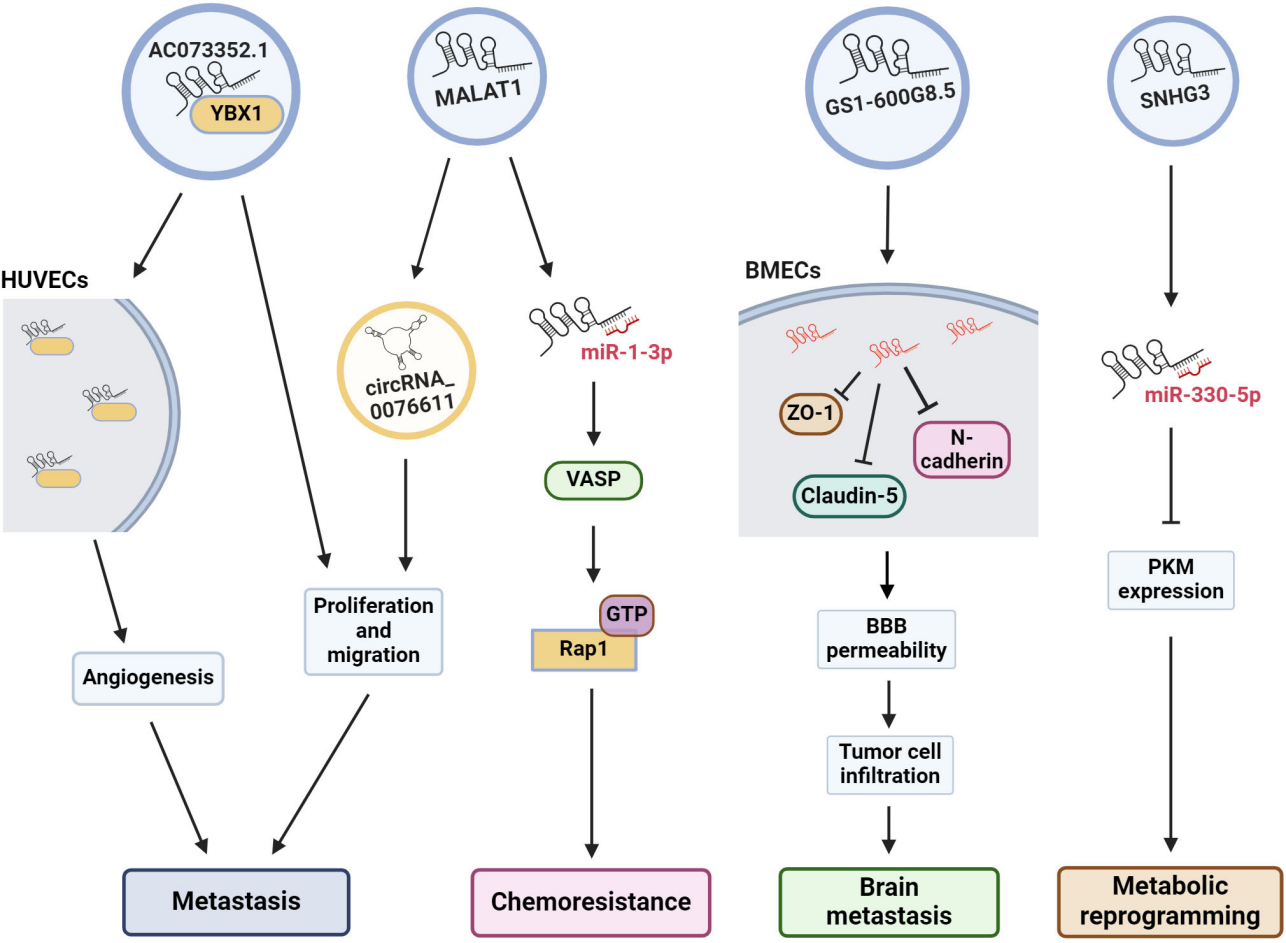
Exosomal lncRNAs are associated to many oncogenic pathways. The illustration shows the effect of some exosomal lncRNAs in the regulation of several cell pathways to induce angiogenesis, metastasis, drug resistance, etc. HUVECs, human umbilical vein endothelial cells; VASP, vasodilator-stimulated phosphoprotein; Rap 1, Ras-related protein 1; BMECs, brain microvascular endothelial cells; BBB, blood-brain barrier; PKM, pyruvate kinase muscle M1/M2.

Another central element for brain metastasis is the integrity of the blood-brain barrier (BBB). Lu and collaborators (2020) (115) determined that exosomes from BC cells disrupt the BBB system, promoting brain metastasis. They established a highly brain metastatic BC cell line (MDABR3, derived from MDA-MB-231 cells) by *in vivo* and *in vitro* selection after 3 rounds of intracardiac injection in mice and subsequent recovery of brain tumors. MDABR3 cells exhibited high levels of migration and invasion *in vitro* and metastatic capacity *in vivo*. Exosomes derived from MDABR3 cells can be taken up by brain microvascular endothelial cells (BMECs), reducing transepithelial/transendothelial electrical resistance (TEER) and increasing permeability in a BBB model, thus promoting invasion of BC cells in the BBB model (**Figure 2**). Using the GEO dataset, the authors selected exosomal lncRNAs associated with the BBB and verified that GS1–600G8.5 was highly expressed in MDABR3 cells and their exosomes than in samples with reduced metastatic behavior. GS1–600G8.5 silencing decreased the BBB permeability induced by MDABR3 exosomes and the infiltration of cancer cells through the BBB. In addition, BMECs treated with GS1–600G8.5-derived exosomes expressed higher levels of tight junction proteins than those treated with exosomes containing GS1–600G8.5 (**Figure 2**) (115).

Angiogenesis is essential for the development of metastasis. Therefore, angiogenesis during cancer progression is another widely studied mechanism. In a recent study, the lncRNA AC073352.1 was identified from a microarray and TCGA database analysis as a novel lncRNA involved in BC metastasis (116). In this study, the authors determined that AC073352.1 was upregulated in BC tissue and was associated with advanced tumor node metastasis (TNM) stage and poor prognosis in BC patients. The overexpression of this lncRNA in MDA-MB-231 and BT549 BC cells (both cell lines with low AC073352.1 expression levels) increased *in vitro* migration and invasion and augmented *in vivo* metastasis in a mouse model. However, AC073352.1 suppression in MCF-7 and MDA-MB-468 cell lines (both with high endogenous AC073352.1 levels) reduced their migration and invasion capabilities (116). They also showed that AC073352.1 promoted BC metastasis and angiogenesis through its binding to YBX1, a transcriptional activator and well-known promoter of metastasis (117). The AC073352.1-YBX1 interaction increases YBX1 stability, and in turn, YBX1 promotes the exosomal internalization of AC073352.1. Moreover, exosomes purified from MDA-MB-231 cells overexpressing AC073352.1 promoted angiogenesis in an *in vitro* tube-formation model using endothelial human umbilical vein endothelial cells (HUVECs) (**Figure 2**) (116).

MALAT1 is another lncRNA found to be important for BC metastasis and doxorubicin resistance (75). Recently, MALAT1 was shown to be highly expressed in MCF-7 cells and their exosomes. Exosomal MALAT1 increases the malignant properties and chemoresistance of BC cells. This effect is mediated by the downregulation of miR-1–3p, which in turn upregulates vasodilator-stimulated phosphoprotein (VASP), resulting in the activation of the Ras-related protein 1 (Rap 1, a member of the RAS oncogene family) signaling pathway (**Figures 1**, **2**) (75), which is well known for promoting invasion, metastasis and chemoresistance (118, 119). MALAT1 is highly expressed in the tissues and serum of BC patients, and higher MALAT1 expression is positively associated with metastasis and TNM stage but negatively associated with overall patient survival (120). Furthermore, MALAT1 promoted the proliferation of MDA-MB-231 and ZR-75–1 BC cells and promoted tumor growth in a xenograft mouse model. Additionally, exosomal MALAT1 from BC cells was able to induce cell proliferation *in vitro* (120). Moreover, MALAT1 induces the expression of circRNA_0076611 and promotes the release of circRNA_0076611 from exosomes of MDA-MB-231 cells (**Figure 2**) (121). CircRNA_0076611 is associated with cell proliferation and migration, and BC patients have been shown to have high levels of this circRNA in serum (121). Another lncRNA involved in metastasis is SNHG3, which has been associated with bone metastasis in BC patients (122). Exosomes from MDA-MB-231 cells containing SNHG3 positively regulate BMP3 expression and bone marrow mesenchymal stem cells (BMSCs), thereby regulating osteogenic differentiation in bone metastasis. Thus, the overexpression of SNHG3 in BC cells may be important for the regulation of osteolytic metastasis (122). All the data described above and summarized in **Table 2**, shows us that lncRNA present several cellular effects that can be both protective and oncogenic in the regulation of BC. The protective effect of XIST expression in various cancers is already well recognized, as well the oncogenic effect of MALAT1. In this way, exosomes are the vehicle for lncRNAs to reach and modulate molecular mechanisms and cellular changes in in distant places.

**Table 2:** Exosomal lncRNAs with metastatic activity in BC.

LncRNA	Status of exosomal lncRNA	Experimental model	Metastatic effect	Reference
XIST	Downregulated	BCC	Induces brain metastasis by stimulating epithelial–mesenchymal transition	Xu et al. (2016) (114)
GS1–600G8.5	Overexpressed	BCC and *in vivo* selection of brain metastatic BCC; BBB *in vitro* model	Induces brain metastasis by increasing bloodbrain-barrier permeability	Lu et al. (2020) (115)
AC073352.1	Overexpressed	BCC and metastatic mice model; *in vitro* tube-formation model using endothelial HUVECs	Induces *in vitro* and *in vivo* migration and invasion and promotes angiogenesis	Kong et al. (2021) (116)

BCC, Breast cancer cells; BC, Breast cancer; BBB, blood-brain barrier; HUVECs, human umbilical vein endothelial cells.

## 5 Other functions of exosomal lncRNAs in breast cancer development: metabolic reprogramming

Previously, we mentioned the many roles of lncRNAs and TDEs in cancer. Several actions of exosomal lncRNAs have been described, particularly in BC. In addition to their role in chemoresistance and metastasis, they can also modulate other cellular processes associated with cellular transformation, including metabolic changes. In this context, MCF-7 and MD-MBA-453 BC cells were treated with exosomes secreted from cultured cancer-associated fibroblasts (CAFs) derived from BC patients. In these assays, CAF exosomes induced the reprogramming of metabolic pathways in BC cells in culture, promoting cell proliferation, decreasing mitochondrial function, and increasing glycolysis, thus modulating metabolic pathways in BC cells (123). This effect is regulated by the lncRNA SNHG3, which is expressed by CAFs and released in exosomes. SNHG3 acts as a molecular sponge for miR-330–5p in cultured BC cells. The suppression of SNHG3 decreases glycolysis and cell proliferation by increasing miR-330–5p, which, in turn, inhibits pyruvate kinase muscle M1/M2 (PKM) expression in cancer cells (**Figure 2**). Accordingly, the inhibition of miR-330–5p by SNHG3 promotes PKM expression, resulting in the inhibition of mitochondrial oxidative phosphorylation and increased glycolytic carboxylation (123). Thus, SNHG3 has been shown to be an important regulator of metastasis, not only by promoting bone metastasis but also by mediating the metabolic transformation of tumor cells. Importantly, the SNHG3 that is released into exosomes from BC cells and CAFs can have cellular effects on numerous types of cells.

## 6 Exosomal lncRNAs as biomarkers for breast cancer patients

**Figure 3:**
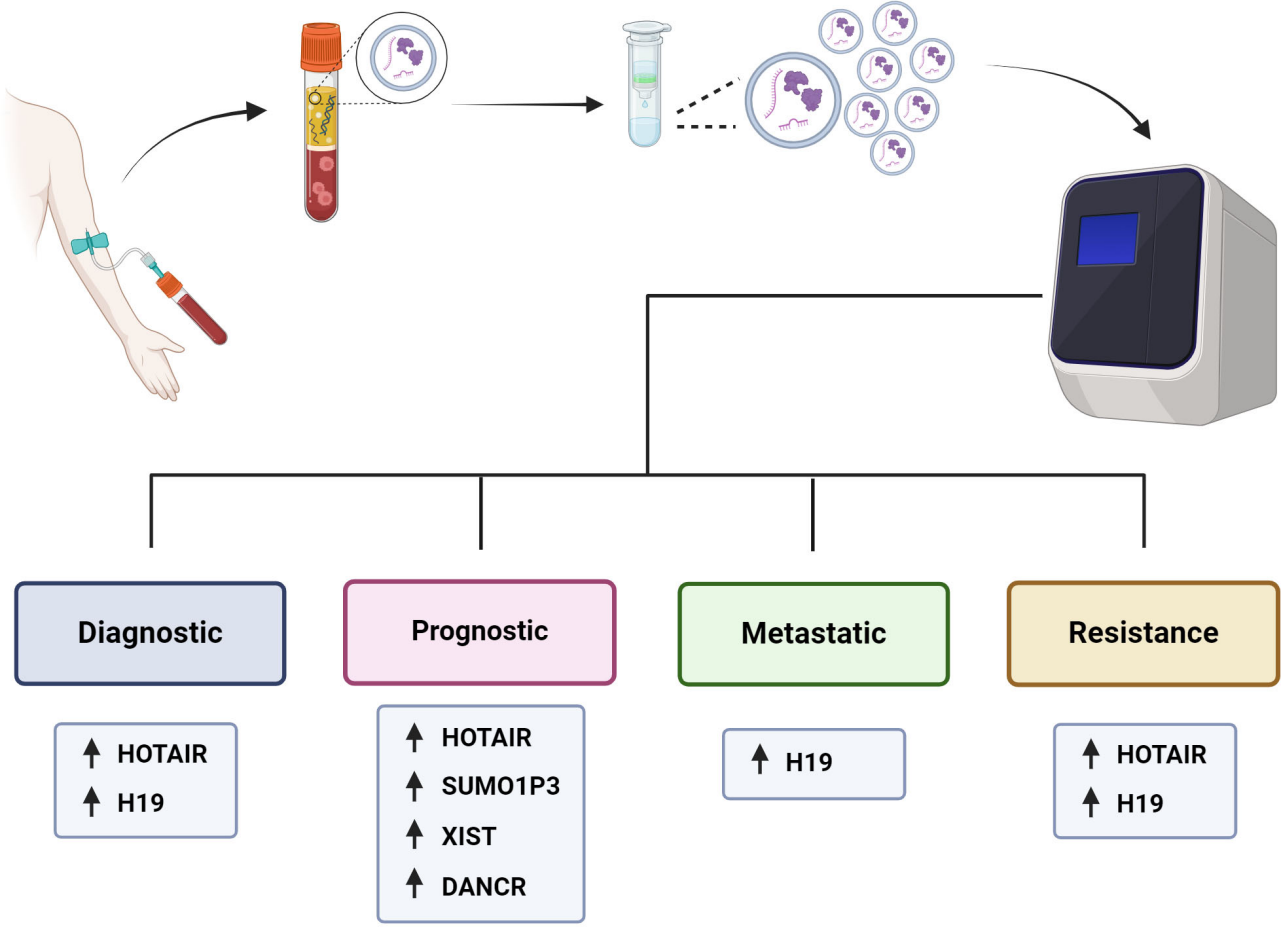
Utility of exosomal lncRNAs as biomarkers of BC. It is outlined how patients with BC would have a peripheral blood sample taken, from which the plasma would be isolated, and the exosomes purified to be analyzed and measure the levels of their components, such as proteins or nucleic acids, including lncRNAs. The levels of exosomal lncRNAs can be measured and used as diagnostic, prognostic, metastatic and drug resistance biomarkers for BC patients.

In cancer research, there is a constant search for biomarkers that will help develop screening tests to achieve more timely diagnoses and better follow-up of BC patients to achieve personalized precision medicine, we summarized the assays made in BC patients in the search for exosomal lncRNAs as biomarkers of BC in the **Table 3** and we outline the use of exosomal lncRNAs as BC biomarkers (**Figure 3**). In this context, the possible use of lncRNA H19 has been studied as a diagnostic biomarker of BC (47). Serum exosomal H19 levels were measured in BC patients, and higher levels were detected in BC patients than in benign breast disease (BBD) patients and healthy controls. Furthermore, when serum exosomal H19 levels were compared between BC patients before and 7 days after surgical removal of the tumor mass, H19 levels were significantly lower in postoperative patients than in preoperative patients. In this manner, the analysis of exosomal H19 levels in serum was shown to be more sensitive and specific than the analysis of cancer antigen 15.3 (CA15.3) and carcinoembryonic antigen (CEA) levels in blood. Moreover, exosomal H19 expression levels are positively associated with lymph node metastasis, distant metastasis, TNM stage, ER status, PR status, and HER2 status (47).

**Table 3:** Exosomal lncRNAs identified as possible biomarkers for BC.

LncRNA	Molecular subtype of BC	Liquid biopsy	Level of exosomal lncRNA	Parameters correlated with levels of exosomal lncRNA	Biomarker type	Reference
HOTAIR	No correlation with the status of any receptor	Serum	High	Correlated with poor neoadjuvant chemotherapy and tamoxifen resistance	Diagnostic and prognostic biomarker for recurrence with poor DFS and OS at 6-year follow-up	Tang et al. (2019) (71)
	HER2+	Plasma	High	Correlation with HER2 status	Prognostic biomarker for HER2 status	Wang at el. (2019) (90)
H19	ER status, PR status, and HER2 status	Serum	High	Correlated with lymph node metastasis, distant metastasis, TNM stage, ER status, PR status, and HER2 status	Diagnostic and metastatic biomarker with better results in combination with CA15–3 y CEA than any of them alone	Zhong at el. (2020) (47)
	Not specified	Serum	High	Predicted BC resistance with a sensitivity and specificity of 75% and 65.2%	Biomarker for doxorubicin resistance	Wang et al. (2020) (74)
SUMO1P3	TNBC status	Serum	High	Correlated with lymphovascular invasion, lymph node metastasis, histological grade and chemoresistance	Prognostic biomarker for TNBC for poor overall survival	Na-Er et al. (2021) (124)
XIST	TNBC	Serum	High	Correlated with poor OS	Prognostic biomarker for recurrence in TNBC at 5-year follow-up	Lan et al. (2021) (125)
DANCR	ER status and HER2 status	Serum	High	Correlated with clinicopathological parameters, including lymph node metastasis, ER status, HER2 status, and TNM stage	Prognostic biomarker for poor OS at 5-year follow-up, with better results in combination with CA15–3 y CEA than any of them alone	Shi et al. (2022) (126)

BC, Breast cancer; TNM, tumor node metastasis; DFS, Disease-free survival; OS, overall survival; CA15–3, cancer antigen 15–3; CEA, Carcinoembryonic antigen.

Na-Er and collaborators (2021) (124) examined the prognostic significance of the serum exosomal lncRNA small ubiquitin-like modifier 1 pseudogene 3 (SUMO1P3) in TNBC. The authors analyzed SUMOP3 levels in BC tissue using the TCGA database. SUMO1P3 was upregulated in BC and correlated with poor survival. Moreover, exosomal SUMO1P3 levels were greater in TNBC patients than in non-TNBC patients, patients with benign breast disease, and healthy people. High exosomal SUMO1P3 levels were correlated with lymphovascular invasion, lymph node metastasis, histological grade and chemoresistance. In addition, patients with high levels of exosomal SUMO1P3 had worse overall survival than patients with low levels of exosomal SUMO1P3. Thus, the authors suggest that exosomal SUMO1P3 might be a prognostic biomarker for TNBC (124). Similarly, Lan and collaborators (2021) (125) proposed exosomal XIST as a biomarker for possible recurrence in TNBC patients. They observed that XIST levels in tumor tissue and exosomal XIST in serum were greater in TNBC patients than in healthy controls. Like those of other lncRNAs, the exosomal levels of XIST decreased after surgical removal of the tumor mass. Furthermore, 5-year follow-up studies revealed greater levels of exosomal XIST in recurrent TNBC patients than in nonrecurrent patients. There was an association between high exosomal XIST levels and poor overall survival, but there was no association with other clinicopathological parameters, such as Karnofsky Performance Status (an assessment tool for functional impairment), sex or age (125).

The lncRNA differentiation antagonizing nonprotein coding RNA (DANCR or ANCR) has been postulated to be a good prognostic biomarker for BC. DANCR has been shown to be involved in the initiation and progression of BC (127, 128). The levels of exosomal DANCR were greater in BC patients than in benign breast disease patients or normal controls, and as expected, exosomal DANCR levels were markedly downregulated in postoperative samples. However, although the diagnostic performance of exosomal DANCR was good, the diagnostic accuracy for BC was better when a combination of exosomal DANCR, CA15.3 and CEA was assessed. In this manner, high exosomal DANCR levels were associated with clinicopathological parameters, including lymph node metastasis, ER status, HER2 status, and TNM stage. Patients with high exosomal DANCR levels had shorter 5-year overall survival, and multivariate analysis of exosomal DANCR levels revealed that this parameter was an independent risk factor for BC (126).

## 7 Conclusion and perspectives

In this review, we describe the importance of exosomal lncRNAs in intercellular communication associated with BC development. The role of lncRNAs in cancer development is well established and the significance of exosomes as mediators of intercellular communication in tumorigenesis has been demonstrated. Particularly, exosomal lncRNAs promote processes such as angiogenesis, invasion, migration, metastasis and chemoresistance, etc, in numerous cancer types, including BC. In this way, the transcriptomic analysis of exosomal lncRNAs is a useful tool in the search for lncRNAs associated with BC tumorigenesis as well in the identification of biomarkers with potential clinical use for the disease.

We have described the significance of exosomal lncRNAs in promoting resistance to drugs such as tamoxifen, trastuzumab, doxorubicin, docetaxel and paclitaxel. Interestingly, it has been observed that resistance to a specific drug can be regulated by more than one exosomal lncRNA, and apparently through different molecular pathways, as we can see in the development of doxorubicin resistance. However, in many cases the intracellular pathways by which some of the lncRNAs induce chemoresistance are not fully described, as in the case of SNHG14. Curiously, the evidence about the chemoresistance induction in sensitive BC cells suggests that the mechanism of drug resistance is dependent on the treatment and not on the molecular profile of the BC cells. For example, the development of chemoresistance to tamoxifen and doxorubicin, in the MCF-7 and MDA-MB-231 cell lines, representatives of different BC subtypes, both cell lines develop resistance to tamoxifen mediated by exosomal HOTAIR and doxorubicin resistance modulated by exosomal H19. Likewise, exosomal lncRNAs not only participate in the development of chemoresistance, but they also modulate metabolic changes in cancer cells as well as several processes associated with metastasis such as proliferation, invasion and angiogenesis. However, the information available regarding the importance of exosomal lncRNAs in the development and progression of BC is still insufficient. There are still many lncRNAs for which the molecular pathways they promote have not been described, and there are surely many more that have not even been identified. Finally, another important aspect is the identification of exosomal lncRNAs and their potential clinical use both as therapeutic targets and as biomarkers of the disease. In this way, there are already approaches and some exosomal lncRNAs have been proposed as diagnostic, prognostic, metastatic and chemoresistance biomarkers. The transfer of lncRNAs mediated by exosomes provides advantages compared with that mediated by cell-free circulating RNA since exosomes provide a more stable environment for measuring macromolecules that reflect tumor characteristics, and these advantages can be exploited to measure different biomarkers that could guide and improve the management of breast cancer. However, exosomal lncRNAs still must be validated for use in clinical trials. Likewise, the most appropriate method for the isolation and analysis of exosomes remains to be validated.

## Author contributions

SB-Z: Writing – review & editing, Writing – original draft, Investigation, Conceptualization. EL: Writing – review & editing. AH-M: Supervision, Writing – review & editing, Resources.
